# How does mental health care perform in respect to service users' expectations? Evaluating inpatient and outpatient care in Germany with the WHO *responsiveness *concept

**DOI:** 10.1186/1472-6963-7-99

**Published:** 2007-07-02

**Authors:** Anke Bramesfeld, Felix Wedegärtner, Hermann Elgeti, Susanne Bisson

**Affiliations:** 1 Department Epidemiology, Social Medicine and Health System Research, Hannover Medical School, Carl Neuberstrasse 1, 30625 Hannover, Germany; 2 Department Clinical Psychiatry and Psychotherapy, Hannover Medical School, Carl Neuberstrasse 1, 30625 Hannover, Germany; 3 Department Social Psychiatry and Psychotherapy, Hannover Medical School, Carl Neuberstrasse 1, 30625 Hannover, Germany

## Abstract

**Background:**

Health systems increasingly try to make their services more responsive to users' expectations. In the context of the World Health Report 2000, WHO developed the concept of health system *responsiveness *as a performance parameter. *Responsiveness *relates to the system's ability to respond to service users' legitimate expectations of non-medical aspects. We used this concept in an effort to evaluate the performance of mental health care in a catchment area in Germany.

**Methods:**

In accordance with the method WHO used for its *responsiveness *survey, *responsiveness *for inpatient and outpatient mental health care was evaluated by a standardised questionnaire. *Responsiveness *was assessed in the following domains: *attention, dignity*, *clear communication*, *autonomy, confidentiality, basic amenities, choice *of health care provider, *continuity*, and *access to social support*. Users with complex mental health care needs (i.e., requiring social and medical services or inpatient care) were recruited consecutively within the mental health services provided in the catchment area of the Hanover Medical School.

**Results:**

221 persons were recruited in outpatient care and 91 in inpatient care. Inpatient service users reported poor *responsiveness *(22%) more often than outpatients did (15%); however this was significant only for the domains *dignity *and *communication*. The best performing domains were *confidentiality *and *dignity*; the worst performing were *choice*, *autonomy *and *basic amenities *(only inpatient care). *Autonomy *was rated as the most important domain, followed by *attention *and *communication*. *Responsiveness *within outpatient care was rated worse by people who had less money and were less well educated. Inpatient *responsiveness *was rated better by those with a higher level of education and also by those who were not so well educated. 23% of participants reported having been discriminated against in mental health care during the past 6 months.

The results are similar to prior *responsiveness *surveys with regard to the overall better performance of outpatient care. Where results differ, this can best be explained by certain characteristics that are applicable to mental health care and also by the users with complex needs. The expectations of *attention *and *autonomy*, including participation in the treatment process, are not met satisfactorily in inpatient and outpatient care.

**Conclusion:**

*Responsiveness *as a health system performance parameter provides a refined picture of inpatient and outpatient mental health care. Reforms to the services provided should be orientated around domains that are high in importance, but low in performance. Measuring *responsiveness *could provide well-grounded guidance for further development of mental health care systems towards becoming better patient-orientated and providing patients with more respect.

## Background

Patients' opinions and views are increasingly being recognized as major indicators of how well health services and health systems are performing, as well as providing guidance for further service improvement [1]. The service users' view is particularly relevant when trying to make health services more responsive to users' expectations. In the context of the World Health Report 2000, WHO developed the concept of health system *responsiveness *as a parameter for a health care system's ability to respond to service users' legitimate expectations of non-medical issues in mental health care [2]. The concept that relates to patient orientation and showing respect for persons in mental health care consists of eight domains: autonomy, confidentiality, communication, dignity, social support, attention, basic amenities and choice. A detailed definition of the domains is presented in table 1[3]:

**Table 1:** Domains as defined in the WHO Responsiveness concept [3]

**Domain**	**Question handles**
Dignity	Being treated with respect
Autonomy	Involvement in decision making
Confidentiality	Confidentiality of personal information
Communication	Listening, enough time for questions, clear explanations
Prompt attention	Convenient travelling distances and short waiting times
Social support	In hospital: visits, having special foods, religious practice
Quality of basic amenities	Cleanliness, space, air
Choice	Seeing a service provider you are happy with

Good *responsiveness *in mental health care is measured by the system's ability to abate the negative side effects that are associated with being mentally ill and undergoing medical treatment. Mental illness and medical treatment affect a patient's sense of autonomy and dignity and cause anxiety and shame. *Responsiveness*, as conceptualised by WHO, aims to strengthen the rights of the individual in the context of the health care system [3,4].

*Responsiveness *in this sense becomes even more important when considering mental illness and mental health care. The characteristics of mental illness and also of some treatments – such as coercive treatment – as well as the stigma still attached to mental health care, make patients even more vulnerable. Therefore, having good *responsiveness *is crucial for mental health care systems. *Responsiveness *is expected to impact positively on health outcomes [5], since it will lower the threshold to seek help early. Beyond this, certain domains such as *communication*, *dignity *and *autonomy *have been shown in studies to positively impact on treatment outcomes [6,7]. Despite the fact that non-medical aspects of care and therapy are often inter-related (particularly in mental health care), *responsiveness *should be considered as an entity on its own. It is one of the three fundamental and independent objectives of health systems as defined in the World Health Report: good health, fair finance and *responsiveness *[2].

In this study we applied the WHO concept of *responsiveness *to a mental health care system for the first time in a standardised way. We thereby attempted to answer the following questions (proposed by WHO as key-questions to *responsiveness *surveys [8]):

• Which aspects of *responsiveness *work well and which less well?

• Are there any differences between the *responsiveness *of inpatient and ambulatory health care services?

• What are the perceptions of *responsiveness *amongst different socio-demographic groups, in particular vulnerable groups, within a country?

• Which *responsiveness *domains are most important to people? Are these ones with good or poor performance? What is the performance of ambulatory and inpatient mental health care in the context of *responsiveness*?

• What are the main reported financial barriers and discrimination to access mental health care?

We applied this concept to a population of service users within a catchment area in the mental health care system in Germany. Psychiatric hospital care in Germany is organised in defined catchment areas. Most outpatient care is provided by psychiatrists in private practice. Patients with more complex illnesses can choose to be treated in psychiatric outpatient departments found in larger cities. They offer more intensive treatment by multi-professional teams. Patients can freely choose where they want to be treated; referral to psychiatric outpatient care is not needed. In case of an acute need for inpatient care, patients are usually confined to being treated their catchment area's hospital. With the exception of a small set fee to be borne by the patient, costs for psychiatric inpatient and outpatient care, including medication, are covered by health insurance companies (98-% of the German population has health insurance [9]).

## Methods

### The concept

WHO developed *responsiveness *as a concept primarily to evaluate general health care systems on a national level. The development of this concept drew on a broad-scale review of literature concerning patient satisfaction and quality of care. Through this review, and also at a meeting of experts in 1999, the eight domains as defined in table 1 were identified [8]. In previous qualitative work we had evaluated the applicability of this concept to mental health care [10]. The concept was proved to suit mental health service users' expectations. However, service users also had additional expectations that were subsumed under a ninth category, namely *continuity *[11].

### The instrument

To measure *responsiveness*, WHO developed and validated a questionnaire. It was used to assess *responsiveness *by population surveys in 60 countries in the Multi Country Service Study (MCSS) [12]. The questionnaire measures *responsiveness *for inpatient and outpatient care in eight domains (although access to *social support *is only assessed in inpatient care) as presented in table 1. *Responsiveness *is measured on a scale ranging from "very good" (one) to "very poor" (five). A more detailed description of the instrument, which meets all classical quality criteria in psychometric testing, and of the MCSS can be obtained from documents available on the internet [8,13,14,14,14]. We tailored the German version of the MCSS questionnaire to suit mental health care by adapting its terminology, adding questions on the additional domain of *continuity *and attaching a section evaluating experiences with day care and hostel care. These are important pillars of mental health care provision. We shortened the time-frame during which experiences with the health care system were assessed from twelve to six months.

To measure the importance of the domains, participants were – in line with the WHO questionnaire – asked to identify the domain they felt was most important to them in mental health care.

Also, in accordance with the WHO approach, barriers to mental health care were assessed. Participants were asked whether they felt they had been treated badly by the mental health care system during the past six months. Various possible reasons for being treated badly (gender, age, etc.) were given. Participants were also asked whether they had decided not to make use of mental health care for financial reasons in the last six months.

State of health was – as in the WHO questionnaire – evaluated with parts of the WHO DAS II [15]. The demographic data assessment was extended to include information on duration of illness, housing situation (e.g., sheltered or independent) and legal guardianship. Finally, the revised questionnaire was tested with experts and service users in respect to its comprehensibility [16].

### The survey

The survey was carried out in the catchment area of the Hanover Medical School's psychiatric departments. The area serves a population of approximately 140,000, living in four districts of the city of Hanover. The city has a total population of 500,000. Between March 1 and June 30, 2006, service users were consecutively recruited in all adult mental health facilities of the catchment area. Private psychiatric practices were not included.

The study was approved by the ethic committee of the Hannover Medical School.

Subjects were recruited after being initially approached by the service staff with regard to their willingness to participate. The criteria for inclusion were: use of complex mental health services in the catchment area during the past six months. "Use of complex services" was defined as making use of social support (e.g., day care, hostel, supported housing) as well as medical support (psychiatrist), or receiving inpatient care during the last six months. In addition to this, participants had to be cognitively capable of following the interview. We chose the criterion " use of complex services" because these service users are the most experienced within the system, generally having greater needs and requiring more intensive care and support. Focusing health care reforms on improving the care of sicker patients with more complex needs has been highlighted as an effective way to improve the performance of the overall system [17].

The interviews were carried out face to face by trained external interviewers. The external interviewers explained the modes of the study once more to the participants and obtained their written consent. Interviews lasted between 45 minutes and one hour. Participants were compensated with 10 €. Interviews were strictly anonymous. To ensure subjects were not accidentally interviewed twice, each record was labelled with a code derived from the participant's name.

Participants were questioned about all parts of the mental health care system that they had experienced during the last six months. However, to prevent interference, only data collected from current inpatient users was used to assess inpatient *responsiveness*. Likewise, only data collected in outpatient care was included in the analyses of outpatient *responsiveness*.

To quantify sampling bias, the socio-demographic properties of the study group were compared to those of patients who had had contact with the mental health care system during a period of twelve months prior to the start of the study.

### Data analysis

Data analysis was done with SPSS for Microsoft Windows. Graphs and figures were produced using Microsoft Excel. In accordance with WHO's approach in the MCSS, *responsiveness *outcomes were dichotomised into *good responsiveness *(combining responses *very good *and *good*) and *poor responsiveness *(combining responses *moderate*, *poor *and *very poor*) [18].

Like WHO, we built an overall *responsiveness *score for inpatient and outpatient *responsiveness*. For this purpose, we averaged the raw values of all domains.

Responses regarding present state of health were dichotomised in a similar way to the *responsiveness *questions.

Differences in *responsiveness *according to socio-demographic characteristics and service style were analysed using parametric tests in cases of normality and non-parametric tests in all other cases. Normal distribution was assessed by the Kolmogorov-Smirnov test and the Shapiro-Wilk test where the sample population was smaller than 50. Differences in *responsiveness *between service styles were analysed for state of health using Mantel-Haenzsel statistics. P-values < 0.05 were considered significant.

## Results

### Study group

312 persons were recruited, 91 in inpatient care and 221 in outpatient facilities (five hostels, two outpatient departments and a company providing sheltered work).

In two of the hostels, the company providing sheltered work and one of the outpatient departments all service users fulfilled the criteria for inclusion. One third of them consented to participation in this study. As we know from analysis of the company providing sheltered work those refusing to being interviewed in the company did not differ by gender (the company had 179 employees, 72% of them were male, and of whom 33% participated) or age. However, many of these service users used several mental health facilities. Thus if they were already interviewed in the sheltered work company they did not sign up for interview in the hostel or outpatient department. While we were able to control that we did not interview someone twice, for data protection reasons, we could not measure how many persons that refused to be interviewed in one facility did so because they were already interviewed in another.

We compared those participants recruited in the outpatient departments for our study with routine data concerning all patients who were treated there the year before (n = 1545). They did not differ by gender, age or duration of illness, or by whether they were living in a hostel or were under legal guardianship. Also participants recruited during inpatient treatment did not differ from those treated there the year before (n = 1055).

Of those participants recruited in outpatient care, 50% had had their last outpatient contact during that last week, 36% between one week and one month ago and 15% between one and six months ago. Two thirds of participants reported an outpatient department as being the location of their last contact with the mental health care system; one third had been to see a practice-based psychiatrist. Of those participants who were recruited in inpatient care, 9% had had to be coerced into being admitted.

Details of the socio-demographic characteristics of the inpatient and outpatient groups are disclosed in table 2.

**Table 2:** Socio-demographic characteristics of the study group

	***Outpatient care users n = 221***		***Inpatient care users n = 91***		***P***
	***n***	***%***	***n***	***%***	
***Gender (male)***	***115***	***52***	***46***	***50.5***	.81
					
***Health self-assessed as poor***	***119***	***53.8***	***55***	***60.4***	.29
					
***Education***					.81
basic level	***74***	***35.7***	***31***	***36.9***	
intermediate level	***79***	***38.2***	***32***	***38.1***	
higher education	***54***	***26.1***	***21***	***25***	
					
***income***					.59
> 500 €	***42***	***24,7***	***24***	***29.3***	
500–1000 €	***86***	***50,6***	***36***	***43.9***	
< 1000 €	***42***	***24,7***	***22***	***26.8***	
					
***Under legal guardianship***	***80***	***36,4***	***22***	***24,2***	**< .05**
					
***Living in hostel or supported housing***	***73***	***33***	***10***	***11***	**< .001**
					
***Working status***					**< .001**
employed	***98***	***45.8***	***22***	***25.6***	
unemployed	***20***	***9.3***	***26***	***30.2***	
retired/disabled	***96***	***44.9***	***38***	***44.1***	
					
***Age (years)***	***45.4***	***SD 11.9***	***43.4***	***SD 16.9***	.24
					
***Duration of illness (years)***	***16.3***	***SD 12.2***	***10.1***	***SD 11.4***	**< .001**

### *Responsiveness *in inpatient and outpatient care

On average, 15% of participants reported negative experiences in outpatient care and 22% in inpatient care. Overall, inpatient care scored worse than outpatient care in every aspect. However, this was only significant for the domains of *dignity *(p = .027) and *communication *(p = .007). This pattern of result did not change when participants who had had to be coerced into being admitted into inpatient care, were excluded. State of health did not contribute to the differences between inpatient and outpatient care (Mantel-Haenszel statistics), except in the case of the domain of *dignity*. Here, inpatients who rated their state of health as good, reported more often poor experiences in the domain of dignity (p = .063).

**Figure 1:**
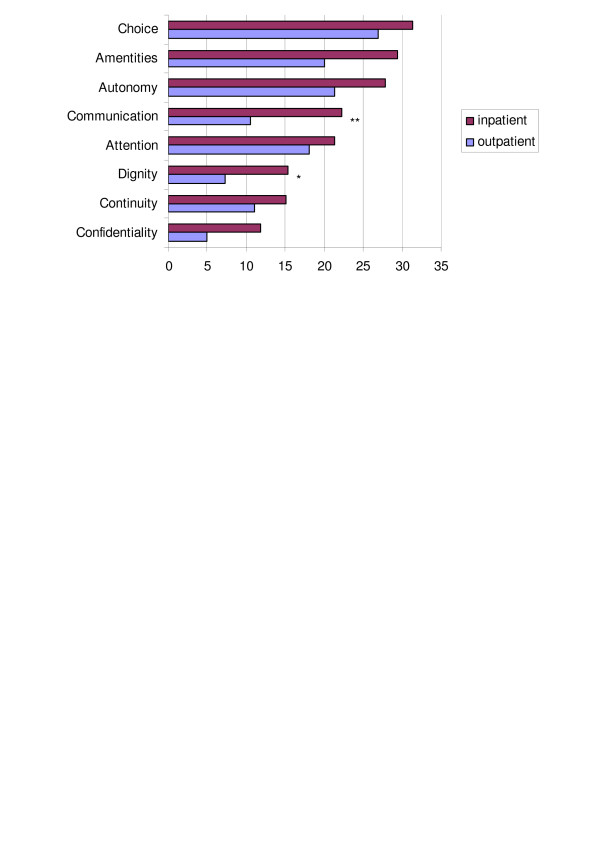
**Percentage of participants rating responsiveness as poor**. *: p < .05, **: p < .005.

Figure 1 shows that the relative ranking of domains was quite similar in both service systems.

Both systems performed best in respect to *confidentiality*. 12% (inpatient) and 6% (outpatient) of users rated this domain as poor. Second best in outpatient care was *dignity *(7%), whilst in inpatient care both *dignity *and *continuity *scored second best, with 15% of participants rating these domains as poor.

Worst performing domains in both service systems were *choice of health care provider *(27% of outpatients and 31% of inpatients) and *autonomy and participation *(21% of inpatients versus 28% of outpatients). *Basic amenities *in inpatient care was rated comparably bad at 29%.

### Importance of domains and performance

**Figure 2:**
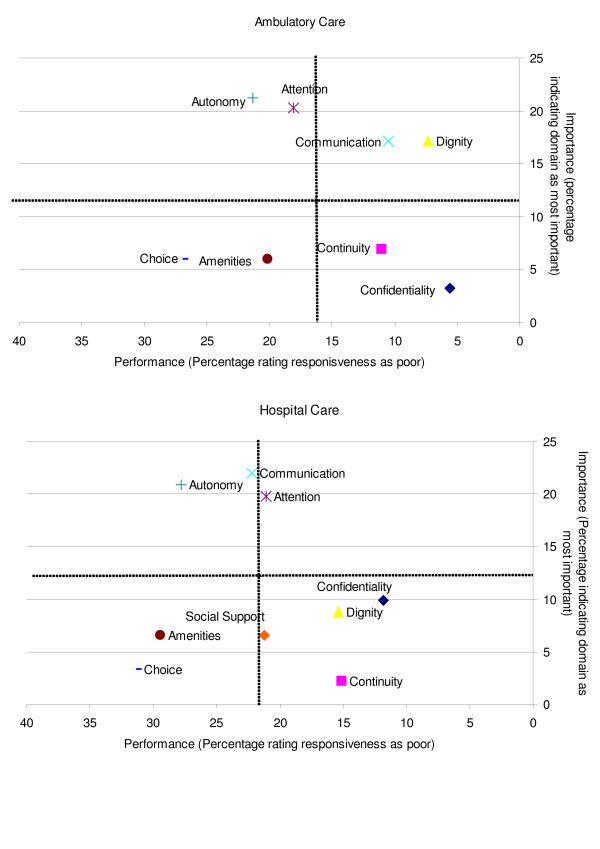
Inpatient and outpatient responsiveness in relation to the importance given to responsiveness domains.

Figure 2 shows the importance of the domains in relation to their performance:

Outpatient care: *autonomy and participation *and *attention *are named by the majority as most important. However, they score amongst the lowest in terms of performance. Only *dignity *and *clear communication *score high in importance and in performance.

Inpatient care: *prompt attention*, which was rated the third most important domain, is the only domain that scores well in both importance and performance (however, the score for performance borders on being not good). *Communication*, which the majority of inpatient service users indicate as most important, performs poorly. *Autonomy *is one of the domains frequently indicated as being most important; however its performance is poor.

### *Responsiveness *in respect to vulnerable groups

The overall inpatient and outpatient *responsiveness *scores were stratified for socio-demographic variables (see tables 3a and 3b) to assess whether specific groups are vulnerable to poorer *responsiveness*:

**Table 3a:** Responsiveness in respect to socio-demographic variables

		***n***	***Mean***	***SD***	***Median***	***Min***	***Max***	***p***
***Gender***								
								
**outpatient**	male	115	1.89	0.48	1.88	1	3.5	.80*
	female	106	1.87	0.47	1.86	1	3.0	
								
**inpatient**	male	46	2.03	0.46	2.0	1	3.3	.89**
	female	45	2.04	0.30	2.0	1.1	3.2	
								
***Present health (self-assessed)***								
**outpatient**	good health	102	1.85	0.45	1.88	1	3.1	.45*
	poor health	119	1.91	0.50	1.88	1	3.5	
								
**inpatient**	good health	36	1.97	0.49	1.94	1.1	3.3	.28**
	poor health	55	2.08	0.43	2.0	1	2.9	

* Mann-Whitney-U-Test, ** T-Test

**Table 3b:** Responsiveness in respect to socio-demographic variables

		*n*	*Rank*	*p*
***Age (quartiles)***				
**outpatient**	0–36 years	43	114.1	.80**
	37–43 years	64	112.5	
	44–53 years	54	104.8	
	> 54 years	60	112.8	
				
**inpatient**	0–36 years	31	46.9	.67**
	37–43 years	15	47.8	
	44–53 years	18	46.5	
	> 54 years	27	43.7	
				
***Duration of illness (quartiles)***				
**Outpatient**	0 – 4 years	39	97.6	.11*
	5–11 years	54	114.4	
	12–22 years	61	124.6	
	> 22 years	66	101.9	
				
**inpatient**	0 – 4 years	37	46.2	.85**
	5–11 years	23	43.4	
	12–22 years	18	42.7	
	> 22 years	11	48.0	
				
***Income***				
**outpatient**	< 500 €	49	96.2	.04**
	500–1000 €	86	92.3	
	> 1000 €	42	73.9	
				
**inpatient**	< 500 €	29	43.3	.63**
	500–1000 €	36	42.7	
	> 1000 €	22	47.1	
				
***Education***				
**outpatient**	basic level	74	115.0	.08*
	intermediate level	79	93.0	
	higher education	54	104.8	
				
**inpatient**	basic level	31	39.8	.02*
	intermediate level	32	51.3	
	higher education	21	33.0	
				
***Working status***				
**outpatient**	employed	98	108.4	.45*
	unemployed	20	122.4	
	retired/disabled	96	103.5	
				
**inpatient**	employed	22	42.8	.99*
	unemployed	26	43.6	
	retired/disabled	38	43.9	

* Kruskal-Wallis test, **Jonckheere-Terpstra test; distribution free test for ordered alternatives in a one-way layout.

#### Outpatient care

*Responsiveness *was rated significantly poorer if people had a lower monthly income. Analysing the duration of illness revealed that in the first three quartiles *responsiveness *worsened the longer a person was ill (p = .03, Jonckheere-Terpstra test). However, service users in the last quartile, who had been ill for more than 22 years, rated *responsiveness *much better. This results in findings which are not significant when all four quartiles are analysed at the same time using Kruskal-Wallis or Jonckheere-Terpstra statistics.

#### Inpatient care

No significant differences in *responsiveness *ratings were found for the variables age, duration of illness, income or working status. However, persons with a basic level of education, as well as those with a university qualification, rated *responsiveness *significantly better than those having an intermediate level of education.

### Barriers to mental health care

23% of all participants reported having experienced discrimination in mental health care for at least one reason. The answer most often given as a reason for discrimination was "other reasons" (15%), followed by "illness" (12%). Taking a closer look at most participants who gave the answer "other reasons" reveals that they seemed to give a response which did not fit the question, e.g., they revealed who was discriminating against them rather than why. Some answers contained paranoic features.

6.5% of study participants reported that on at least one occasion in the past six months they did not ask for mental health care because they felt they could not financially afford it.

## Discussion

In this study we tried to measure the *responsiveness *of mental health care by the example of a regional mental health care system in a larger German city. The study group can be considered representative of service users in psychiatric inpatient care and of service users using complex services in urban areas of Germany.

It is interesting to compare the ratings of *responsiveness *in mental health care with data on general health care *responsiveness*. Within the framework of the MCSS, WHO assessed the *responsiveness *of the general health system in Germany. For this purpose, a sample of the German general population (n = 1123) was surveyed using comparable methods. 698 persons revealed contact to outpatient care and 96 to inpatient care [19]. Our findings are discussed in the light of this prior study. By doing so, we attempt to answer the key questions which were proposed by WHO for *responsiveness *surveys.

### Which aspects of *responsiveness *work well and which work less well?

*Confidentiality *is the best performing domain in inpatient and outpatient care. This finding is in line with the WHO results for the general health care system [19]. Except for cases of severe violation of data protection, patients do not know whether their personal information is handled confidentially or not. However, the general health system and the surveyed mental health system seem to be able to build an atmosphere of trust and promote confidentiality. In fact, standards of data protection in psychiatry are very high. Without a patient's written consent, no case related information can be passed on except to the referred service.

Also, the domains *dignity *and *access to social support *while in inpatient care perform well both in the German MCSS and also in our study. This is not the case for *choice of health care provider *and *quality of basic amenities*. Unlike in general health care, these domains are among the worst performing ones in inpatient care [19]. The relatively poor performance of *basic amenities *might reflect the fact that rooms and furniture on psychiatric wards often do not meet the standards that patients have experienced in other clinics. Also to be taken into account is the fact that psychiatric patients often spend (live) many weeks on ward while being in better physical shape than most average medical or surgical patients. Thus, expectations of their surroundings might be higher.

In mental health care, there is indeed less opportunity for free *choice *in terms of health care provider. This is not only due to the fact that some patients have to undergo coercive treatment but more so due to the scarcity of facilities, the lack of need for competition between facilities for service users and due to the lack of information about alternative services and treatments [20]. This often minimises the choices a patient has and as such, the patient is forced to take whatever is available or not to seek help at all. Poor opportunities for *choice *are aggravated by the policy of many service providers that for therapeutically reasons do not support service users to change therapists if they do not like the one they are with.

*Autonomy *does not perform very well in mental health care. The same result is found in *responsiveness *surveys that focus on general and primary health care [18]. The difficulties involved in letting patients participate in decisions, thereby strengthening their *autonomy*, is thus not a specific mental health care problem (which, if it were, would be explained by the nature of mental illness). Rather, it seems to be a general problem in medical care that there is still a strong information gradient between provider and service users; paternalistic self-images still persist and consumer empowerment is a challenge that needs to be worked on [21].

### Are there any differences between the *responsiveness *of inpatient and ambulatory health care services?

Only in the domains *dignity *and *clear communication *do statistics differ significantly between inpatient and outpatient care. In both the global and the German data, the MCSS revealed poorer ratings for inpatient care in all domains, but failed, however, to report on statistical significance [19,18]. The difference in the *dignity *rating between mental health inpatient and outpatient care is mediated by state of health. Inpatients who feel healthy are more critical in respect to *dignity *which is one of the domains considered most important in mental health care by participants. The difference in ratings among healthy patients might be explained by a kind of "selection effect": the healthier the inpatients become, the more they will question the need for putting up with life on a hospital ward. However, attaining preliminary discharge requires much effort and a lot of arguing with the therapist. In contrast, in outpatient care, patients who feel healthy and who are not content with *dignity *and respect within their treatment, will simply not keep their next appointment. Also, people rating their health as poor might be more convinced about the need for care and, therefore, will probably adjust their expectations about being treated with *dignity*.

Differences in ratings for clear *communication *might be explained by a greater need in hospital for receiving information that a patient can fully understand. This need might be related to the often unfamiliar situation of inpatient care and a patient's greater dependency under these conditions. In addition, relationships in outpatient care are usually long-lasting. Therefore, after a while, most basic questions have probably been discussed.

### What are the perceptions of *responsiveness *among different socio-demographic groups, in particular vulnerable groups?

The German MCSS found that inpatient *responsiveness *was perceived as worse by all vulnerable groups, i.e., the elderly, the indigent, the less educated and the sicker. In outpatient care, *responsiveness *was rated worse by the less educated, the sicker and the indigent.

However, our findings in mental health care differ from those of the MCSS: whilst outpatient care was perceived differently depending on education and income, we did not find a difference in respect to state of health. Our study group was probably more homogeneous in respect to health (mostly long-term ill and in need of complex services) than the MCSS general population group. As *responsiveness *was rated worse the longer a patient was ill, astonishingly, those who had been ill for a very long time rated *responsiveness *quite well. One hypothesis for this behaviour is that people might lower their expectations during the course of an illness. However, if this were the case, this trend should also have been shown in the third quartile of patients who had been ill for 12 to 22 years. Another explanation is, that those who have been ill for more than 22 years have experienced psychiatric care both before and at the beginning of mental health care reforms 30 years ago. Therefore, they have indeed experienced very low standards of care as a means of comparison.

Other than education, the perception of *responsiveness *did not differ for socio-demographic characteristics in inpatient care. We do not have a convincing explanation for the relationship between education and *responsiveness*, particularly when considering that in inpatient care, those with an intermediate level of education perceive *responsiveness *worse than in outpatient care where it is perceived as better. We believe more research might be useful to clarify the relationship between education and experiences with mental health care.

Although this was not the case in the MCSS mental health inpatient *responsiveness *did not differ much according to socio-demographic variables. This finding can be explained by the fact that inpatient treatment in psychiatry is more uniform than outpatient care or non-psychiatric treatment. For example, the Hanover Medical School's psychiatric departments do not charge higher amounts for privately insured patients as they do not offer privileged services to them. Also, psychiatric inpatient treatment includes group activities in which usually everyone – regardless of status or severity of mental illness – is entitled to participate. Finally, it might be that those who are better off, both financially and in terms of health, are able to draw on social or other resources during outpatient care that are not available to them in the uniform and restricted atmosphere of inpatient care.

### Which *responsiveness *domains are most important to people? Are these the ones with good or poor performance results?

Those domains rated less often as being important should not be interpreted as marginal. In most cases, they are those that perform relatively well, as is the case with *dignity*, *social support*, *confidentiality *and *continuity*. Also the characteristics of a domain such as *continuity *(added particular as a new domain to the concept) to reveal its quality primarily in a longitudinal perspective might have added to rating it as less important. As also the qualitative research into mental health system responsiveness has shown, *continuity *is a relevant domain however, compared to other domains such as *autonomy *not prominent [10]. At the same time, the ratings of a domain assessed as being most important might in fact be negatively influenced by poor performance.

There is a cluster of three domains rated by the majority as most important: *attention*, *autonomy *and *communication*. *Clear communication *is valued much higher in inpatient care for reasons discussed above and is related to inpatients being more often in a situation that is unfamiliar to them and them therefore having greater dependency.

*Prompt attention *seems to be a core expectation in general health care, as shown by the MCSS. The high rating of *autonomy *– although this is also known from other medical sectors [22] – might have a specific meaning for mental health care. Cognitive constraints are frequent, denial of illness and refusal of treatment too. Also, the possibility of coercive treatment exists. All these aspects lead to a more paternalistic approach than in other medical specialities [23]. The specific desire of mental health service users to be involved in mental health care decisions has been highlighted in other studies too [24,23]. Qualitative exploration of service users' expectations in psychiatry shows that the meaning of *autonomy *does not only imply the idea of shared decision making but also implies transparency and involvement in report writing. This is not a claim stemming from patients simply being in denial about their illness. Patients accept that there are certain mental states where they are not capable of making all decisions. However, the more they recover, the more they want to be involved [10].

It is of cause for concern that *autonomy *and *attention*, indicated so often as most important, do not perform well either in outpatient or inpatient care in the study catchment area. The poor performance of *autonomy *is probably not only restricted to the catchment area surveyed. More *autonomy *and *participation *is also a general claim made by service user organisations [25].

### What are the main reported financial barriers and issues of discrimination with regard to obtaining access to mental health care?

The MCSS found that in 2001, 5% of the German population did not ask for health care because of financial reasons. This figure was slightly higher in our study population. Because our sample population included only those who had finally succeeded in entering the mental health care system despite financial barriers, no precise statement about the real impact of financial barriers can be made.

Also, the investigation into possible issues of discrimination proved to be difficult in the context of this study. The responses given naming "other causes" as reasons for discrimination indicate that the question was misunderstood by quite a number of participants. We have concluded that the *responsiveness *questionnaire and the format of this study are not appropriate for assessing barriers to mental health care.

## Conclusion

*Responsiveness *as a parameter for the quality of health care does indeed provide a refined picture of inpatient and outpatient performance in mental health care. Even if only the views of service users with complex service needs are considered, results of this study can be transferred to all users and provide guidance for further development and improvement in mental health care [17].

Domains that are rated high in importance and poor in performance should be given priority and measures should be implemented to improve services. Such domains include *prompt attention *and *autonomy and participation in decisions *both in inpatient and ambulatory mental health care. There are indications to show that including cognitively impaired persons and those who deny their illness in decision making may lead to better attitudes towards mental health treatment and compliance [23]. Methods to increase *autonomy *and participation of mental health service users include shared decision making and improvement in the transparency of mental health reports. Also, models used for other chronic illnesses and diseases, such as diabetes, rheumatoid arthritis and asthma, that purposefully train patients to become experts on their illness [22] and encourage self-management in a structured way, should be explored for ways in which this could be transferred to mental health care. All these measures not only strengthen a patient's participation and control over treatment, but also go hand in hand with increasing the specific knowledge and information about their illness. Thus, there is a strong link here to the domain of *communication*. Good information and clear *communication *seems particularly difficult to attain for persons who have mental problems [26].

*Responsiveness *as a parameter of health system performance provides a structured way to evaluate mental health services in the areas of patient orientation and treating a patient with respect. However, the instrument used in this study is much too complicated and in-depth for routine use. It is planned that the instrument will soon be revised and shortened to make it into a short, self-administrable and easy to understand tool that can be realistically applied in real clinical life. This would provide the opportunity for routine evaluation and for benchmarking service systems with results being fed back to service providers.

Close to 30 years of mental health care reforms in Germany has led to quite a number of community-orientated service provisions. However, motivation for reform has, for some reason, slowed down in recent years [27]. The concept of *responsiveness *can offer new controllable guidelines for service development and can help better achieve meeting patients' expectations and strengthening them within the system.

## Competing interests

The author(s) declare that they have no competing interests.

## Authors' contributions

AB conceived and proposed the study, interviewed patients, analysed the data and drafted the manuscript.

FW helped with recruitment, interviewed patients, processed and analysed data and drafted the manuscript.

HE organised recruitment and contributed to the design of the study.

SB advised on statistical analysis and drafted the manuscript.

All authors have read and approved the final manuscript.

## Pre-publication history

The pre-publication history for this paper can be accessed here:


